# Draft Genome Sequence of Enterobacter sp. Strain UCD-UG_FMILLET (Phylum *Proteobacteria*)

**DOI:** 10.1128/genomeA.01461-14

**Published:** 2015-01-22

**Authors:** Cassandra L. Ettinger, Walaa M. Mousa, Manish N. Raizada, Jonathan A. Eisen

**Affiliations:** a Genome Center, University of California Davis, Davis, California, USA; b Department of Plant Agriculture, University of Guelph, Guelph, Ontario, Canada; c Department of Pharmacognosy, School of Pharmacy, Mansoura University, Mansoura, Egypt; d Department of Evolution and Ecology, University of California Davis, Davis, California, USA; e Department of Medical Microbiology and Immunology, University of California Davis, Davis, California, USA

## Abstract

Here, we present the draft genome of Enterobacter sp. strain UCD-UG_FMILLET. This strain is an endophyte isolated from the roots of finger millet, an Afro-Indian cereal crop. The genome contains 4,801,411 bp in 53 scaffolds.

## GENOME ANNOUNCEMENT

Enterobacter species are generally known as aerobic Gram-negative rod-shaped bacteria. Enterobacter species have been found as plant growth promoting endophytes and as antagonists to fungal plant pathogens (1–3). As part of an ongoing project investigating the antifungal properties of endophytic bacteria, Enterobacter sp. strain UCD-UG_FMILLET was isolated from the roots of the Afro-Indian cereal crop, finger millet (*Eleucine coracana*, imported commercial Indian variety), at the University of Guelph, Canada during August, 2012.

Finger millet seeds were planted under semi-hydroponic conditions in 22.5 liter pales placed in a field (GPS, 43°39′N, 80°25′W Arkell Field Station, Arkell, ON, Canada) during the summer of 2012. Roots were surface sterilized as previously described (4). Tissues were ground in lysogeny broth (LB) liquid medium in a sterilized mortar and pestle, and 50-µL suspensions were plated. Colonies were purified by repeated streaking on fresh medium. Genomic DNA was extracted at Guelph using a GenElute Bacterial Genomic DNA kit (NA2110-1KT, Sigma), then ethanol precipitated before shipment to the University of California, Davis, for library preparation, sequencing, and analysis. Illumina paired-end libraries (read length, 250 bp) were made using a Nextera DNA sample preparation kit (Illumina) and were sequenced on an Illumina MiSeq.

A total of 9,534,168 paired-end reads were produced; after quality trimming and error correction, 9,320,558 high-quality reads remained. Sequence processing and assembly were performed using the A5 assembly pipeline (version A5-miseq 20140604) following the workflow described by Dunitz et al. (5, 6). The assembly resulted in 74 contigs contained in 53 scaffolds (minimum, 441 bp; maximum, 838,849 bp; *N*_50_, 236,194 bp). The final assembly contained 4,801,411, had a GC content of 55.76%, and median coverage of 451×. Genome completeness was assessed using Phylosift software (v1.0.1), which searches for 37 highly conserved single copy marker genes, all of which were found in this assembly (7, 8).

Automated annotation was completed using the RAST server (9). Enterobacter sp. strain UCD-UG_FMILLET contains 4,533 predicted protein-coding sequences and 112 predicted non-coding RNAs. Previous sequencing of the 16S rRNA gene identified this isolate to be a representative of Enterobacter and a full-length (1,789 bp) 16S rRNA gene sequence was obtained from the RAST annotation to attempt to more precisely determine the taxonomic identity of this isolate. A phylogenetic tree was constructed using FastTree 2 by aligning this sequence to 16S rRNA gene sequences from 46 Enterobacter isolates from the Ribosomal Database Project (RDP) and an archaea outgroup (http://dx.doi.org/10.6084/m9.figshare.1245106) (10, 11). We were unable to identify the taxonomy of the isolate to the species level, as it did not cluster into a distinct clade with a specific Enterobacter species. The 16S sequence of Enterobacter sp. strain UCD-UG_FMILLET is greater than 99% identical to the 16S sequences of several Enterobacter species; therefore, we are unable to assign a species name to this isolate.

### Nucleotide sequence accession numbers.

This whole-genome shotgun project has been deposited at DDBJ/EMBL/GenBank under the accession no. JRJC00000000. The version described in this paper is version JRJC01000000. The raw Illumina reads are available at ENA/SRA accession no. PRJEB7722 (ERP008661).
